# ART treatment costs and retention in care in Kenya: a cohort study in three rural outpatient clinics

**DOI:** 10.7448/IAS.16.1.18026

**Published:** 2013-01-02

**Authors:** Bruce A Larson, Margaret Bii, Sarah Henly-Thomas, Kelly McCoy, Fredrick Sawe, Douglas Shaffer, Sydney Rosen

**Affiliations:** 1 Center for Global Health and Development, Boston University, Boston, MA, USA; 2 Department of International Health, School of Public Health, Boston University, Boston, MA, USA; 3 Kenya Medical Research Institute, Kericho, Kenya; 4 Castalia Advisors, Washington, DC, USA; 5 United States Army Medical Research Unit-Kenya, Walter Reed Project, Kericho, Kenya; 6 Health Economics and Epidemiology Research Office, Department of Internal Medicine, School of Clinical Medicine, Faculty of Health Sciences, University of the Witwatersrand, Johannesburg, South Africa

**Keywords:** HIV, Kenya, antiretroviral therapy, cost analysis, retent

## Abstract

**Introduction:**

After almost 10 years of PEPFAR funding for antiretroviral therapy (ART) treatment programmes in Kenya, little is known about the cost of care provided to HIV-positive patients receiving ART. With some 430,000 ART patients, understanding and managing costs is essential to treatment programme sustainability.

**Methods:**

Using patient-level data from medical records (*n=*120/site), we estimated the cost of providing ART at three treatment sites in the Rift Valley Province of Kenya (a clinic at a government hospital, a hospital run by a large agricultural company and a mission hospital). Costs included ARV and non-ARV drugs, laboratory tests, salaries to personnel providing patient care, and infrastructure and other fixed costs. We report the average cost per patient during the first 12 months after ART initiation, stratified by site, and the average cost per patient achieving the primary outcome, retention in care 12 months after treatment initiation.

**Results:**

The cost per patient initiated on ART was $206, $252 and $213 at Sites 1, 2 and 3, respectively. The proportion of patients remaining in care at 12 months was similar across all sites (0.82, 0.80 and 0.84). Average costs for the subset of patients who remained in care at 12 months was also similar (Site 1, $229; Site 2, $287; Site 3, $237). Patients not retained in care cost substantially less (Site 1, $104; Site 2, $113; Site 3, $88). For the subset of patients who remained in care at 12 months, ART medications accounted for 51%, 44% and 50% of the costs, with the remaining costs split between non-ART medications (15%, 11%, 10%), laboratory tests (14%, 15%, 15%), salaries to personnel providing patient care (9%, 11%, 12%) and fixed costs (11%, 18%, 13%).

**Conclusions:**

At all three sites, 12-month retention in care compared favourably to retention rates reported in the literature from other low-income African countries. The cost of providing treatment was very low, averaging $224 in the first year, less than $20/month. The cost of antiretroviral medications, roughly $120 per year, accounted for approximately half of the total costs per patient retained in care after 12 months.

## Introduction

Almost 10 years after public sector provision of antiretroviral therapy (ART) began in many African countries, better information on the costs to healthcare providers for delivering medical care to patients on ART is still needed. Such costs obviously include not only the cost of antiretroviral medications but also the cost of laboratory tests, non-ART medications, medical staff providing care and fixed costs at the site. At a time when donor budgets have tightened in response to the persistent global economic crisis, though demand for treatment continues to grow, better information on the costs and structure of costs of delivering AIDS care and treatment in different settings would help policy makers and funding agencies allocate future resources more efficiently, thereby promoting the long-term sustainability of ART treatment programmes in Africa's low-income countries [1–4].

A handful of studies have been published on the costs of ART in Africa. In one of the earliest studies in South Africa in 2007, the average cost per patient treated at a large public sector clinic during the first year on ART was $756 [5]. Since then, other estimates have been made for South Africa and a few low-income countries in Africa, including Ethiopia, Nigeria, Uganda and Zambia [6–11]. However, despite the fact that Kenya has been one of the largest recipients of funds from the US President's Emergency Plan for AIDS Relief (PEPFAR), second only to South Africa in funding received, as well as a major recipient of support from the Global Fund to Fight AIDS, Tuberculosis and Malaria (GFATM), a recent literature review of ART cost studies did not find a single published estimate for Kenya [12–14].

We present here the results of a micro-costing analysis of the provision of ART at three treatment sites in the Rift Valley Province of Kenya. The study sites, which are all supported by PEPFAR, provide free outpatient ART, serve predominately rural population and are typical of rural clinics where ART is delivered to thousands of patients throughout Kenya.

## Methods

### Study sites and sample selection

The study was conducted in Kenya, where approximately 430,000 people were on ART at the end of 2010 [15]. Data were collected at three sites. Site 1 is an HIV clinic within a government-run district hospital compound located in Kericho, Kenya. It is a typical district hospital with a large public-sector HIV treatment programme. Site 2 is a private hospital owned and managed by a multinational agricultural company. The company hospital is located a few kilometres away from the district hospital, which would be the likeliest substitute location for HIV treatment for workers if the company hospital did not offer it. Although the company hospital has access to better resources than the public hospital, it follows the same treatment guidelines and procedures as the public sector hospital. Site 3 is an HIV clinic in a mission (faith-based) hospital with minimal resources. Sites 1 and 2 have received PEPFAR support since 2004 and Site 3 since 2005.

The retrospective, cohort-based, micro-costing approach used in previously published studies in South Africa is followed here [5,8]. Under Kenya Ministry of Health guidelines for ART in use at all our study sites in 2007, adult, non-pregnant patients were eligible to initiate ART if they had a CD4 cell count ≤200 cells/mm^3^, were in WHO Stage 3 and had a CD4 count below 350, or were in WHO Stage 4 regardless of CD4 count. The preferred first-line antiretroviral (ARV) regimen in Kenya until 2010 was stavudine (d4T) or zidovudine (AZT), plus lamivudine (3TC) and efavirenz (EFV) or nevirapine (NVP). CD4 counts were required at initiation and every six months thereafter, but viral load tests were not required.

At each site, we selected the first 120 adults initiated on ART as of January 2007 who met study eligibility criteria: at least 18 years of age; not transferred formally to another treatment site during the 12 months following treatment initiation; not pregnant; and patient files available for review.

The Boston University Medical Campus and the Kenya Medical Research Institute provided ethical review.

### Resource utilization and primary outcome

For each study subject, we reviewed patient medical records to determine quantities of ARV medications, non-ARV drugs, laboratory tests, and clinic visits utilized by each patient in the 12 months following ART initiation.

Medical record data were also used to assign each sampled patient an outcome of “in care” or “not in care” 12 months after initiating ART. Not in care included deaths and losses to follow-up, defined as being more than 3 months late for the scheduled appointment closest to the end of month 12.

### Unit costs, fixed costs, and total costs

Following the same approach used in previous patient-level costing analyses [5], we estimated the total cost of outpatient care and treatment for each patient in the study sample, from the provider's perspective, over the 12 months after ART initiation. All resources used by the provider for outpatient care during the 12 month period, even if the resource cost was borne by another programme or organization, were included in costs. Costs incurred elsewhere, above the level of the site evaluated here, such as government costs of oversight, training, or research, or fund-raising by the NGO network outside of Kenya for the NGO site, were excluded from the analysis.

For each patient, total cost is the sum of fixed costs and variable costs, where variable costs are the quantity of each type of resource utilized for each patient multiplied by its unit cost. Costs of ARV drugs were obtained directly from the PEPFAR programme operating in the region that sourced ARVs for the treatment sites. Unit costs of non-ARV drugs and laboratory were estimated with assistance from the accounting staff at each site from invoices and itemized expense reports. A unit cost per patient interaction with medical staff during a clinic visit was estimated by dividing the salaries and benefits paid for each level of staff by the estimated number of patient interactions per month completed by each level.

All costs incurred by the sites not directly attributable to individual patients were considered fixed costs. Fixed costs included annual site-level costs for supplies, such as cleaning and general office supplies, maintenance for equipment and buildings, insurance, utilities, and staff not interacting directly with patients (cleaners, drivers, filing clerks, accountants, etc.). Fixed costs also included equipment, vehicles, and buildings purchased and used at the site, which were annualized based on expected service lives, a 7% real discount rate, and inflated from the purchase year up to the base year for the analysis (2009).

Following the approach used in previous studies [5], we allocated total annual fixed costs on the basis of the total number of patients enrolled in ART and pre-ART care to allocate fixed costs per patient-month in care. Each study subject was then assigned a fixed cost equal to that subject's number of months in care times the average cost per patient-month in care. Costs were converted from Kenya shillings at an exchange rate of KES 72.2/USD and are reported in 2009 USD.

## Results

Table 1 describes the sites, cohorts, and patient outcomes. Site 1, based at a district hospital, was three times larger than the other two sites. At all sites, median CD4 cell count at baseline was well below 200 cells/mm^3^. Sites 1 and 3 used the same first line regimen, d4T/3TC/NVP, while Site 2 used d4T/3TC/EFV. One patient was switched to second line treatment during the 12 months after ART initiation at Site 3, and none at Site 1 or Site 2.

**Table 1:** Description of study sites and outcomes (2009)

	Site 1 (n=120)	Site 2 (n=120)	Site 3 (n=120)
Site characteristics			
Location	Town	Rural	Rural
Organization structure	Government district hospital	Private company hospital	Mission hospital
Location of ART services	ART clinic within hospital compound	General outpatient ward	ART clinic within hospital compound
Number of ART patients at site	2,566	821	826
Primary first-line regimen	D4T-3TC-NVP	D4T-3TC-EFV	D4T-3TC-NVP
Patient outcomes at 12 months (%)			
In care	98 (82)	96 (80)	101 (84)
Died	7 (6)	1 (1)	7 (6)
Lost to follow-up	15 (12)	23 (19)	12 (10)
Median baseline CD4 count (all study subjects)	98	143	117

The three sites achieved similar outcomes, with 82%, 80%, and 84% of patients remaining in care at 12 months (Table 1). Among those not remaining in care, 24% were known deaths, while the rest were lost to follow-up, a category that likely includes some unreported deaths and transfers as well as true losses to care.

As reported in Table 2, the average cost per patient initiated on ART was $206, $252, and $213 at Site 1, 2, and 3, respectively. ARV drugs accounted for the largest share of total costs at each site (50%, 44%, and 49%), with the remaining costs split among non-ARV drugs (16%, 11%, 12%), lab tests (14%, 14%, 15%), personnel providing patient care at clinic visits (9%, 12%, 12%), and fixed costs (11%, 18%, 13%).

**Table 2:** Average costs disaggregated by input categories (USD 2009)

Input Categories	Site 1 USD (%)	Site 2 USD (%)	Site 3 USD (%)
All patients initiated on ART at 12 months	n=120	n=120	n=120
Drugs – ARV	103.5 (50)	112.0 (44)	105.8 (49)
Drugs – non-ARV	33.4 (16)	28.0 (11)	22.2 (10)
Lab tests	28.5 (14)	36.2 (14)	31.1 (15)
Visits	18.8 (9)	29.2 (12)	25.8 (12)
Support services	0.1 (0)	0.2 (0)	0.9 (0)
Fixed costs	22.4 (11)	46.6 (18)	27.9 (13)
Total	206.7 (100)	252.2 (100)	213.8 (100)
Only patients retained in care at 12 months	n=98	n=96	n=101
Drugs – ARV	117 (51)	127 (44)	119 (50)
Drugs – non-ARV	36 (15)	31 (11)	24 (10)
Lab tests	31 (14)	43 (15)	34 (15)
Visits	20 (9)	33 (11)	28 (12)
Support services	0 (0)	0 (0)	1 (0)
Fixed costs	25 (11)	53 (18)	32 (13)
Total	230 (100)	288 (100)	237 (100
Only patients not retained in care at 12 months	n=22	n=24	n=19
Drugs – ARV	42.2 (40)	49.6 (44)	38.3 (43)
Drugs – non-ARV	23.9 (23)	14.5 (13)	14.7 (17)
Lab tests	16.9 (16)	12.8 (11)	13.9 (16)
Visits	12.1 (12)	14.4 (13)	11.8 (13)
Support services	0.3 (0)	0.0 (0)	0.7 (1)
Fixed costs	9.1 (9)	21.9 (19)	8.9 (10)
Total	104.4 (100)	113.2 (100)	88.3 (100)

Average cost per patient for the subset of patients remaining in care at 12 months was $230, $288, and $237, respectively, for each site. The distribution of costs across input categories for the subset of patients retained in care was essentially the same as average costs for all patients summarized above. The higher costs at Site 2 were due to higher fixed costs per patient-month in care and a larger number of laboratory tests per patient-year. Site 2 also averaged slightly more visits per patient in care (9.5 over the 12 month period) than did Site 1 and 3 (8.3 and 8.9, respectively).

Average cost per patient for the subset of patients not in care at 12 months was $104 at Site 1, $113 at Site 2, and $88 at Site 3. On average, these patients received 5, 6, and 4 months of care after initiating ART, with fewer resources utilized largely due to the shorter period of care.

Average costs per patient initiated on treatment and for the subset of patients retained in care at 12 months are fairly similar across the three treatment sites, even though Site 1 (the government site) had substantially larger numbers of patients receiving ART. This result reflects the fact that variable costs for each patient were the major share of total costs, variable costs are driven by the quantity of resources used in patient care, and the small sites had smaller fixed costs.

Average costs per patient initiated on ART divided by the proportion retained in care can be interpreted as the average cost to produce a patient retained in care at 12 months [5]. Based on the average cost information reported in Table 2 and retention rates reported in Table 1, the average costs to have a patient retained in care at 12 months is $252, $315, and $260, respectively.

## Discussion

At three healthcare facilities in rural Kenya, at least 80% of patients who initiated ART in 2007 remained in care 12 months after initiation. This retention rate matches or exceeds the median reported by a review of studies from 2007–2009 from across the continent, which estimated that, throughout Africa, median retention in care 12 months after treatment initiation was 79.4%, with a range of 55–93% [16]. It is also better than the average of 72.5% (range 62–85%) achieved by all seven studies from Kenya included in that review and in a previous one [16,17].

The cost of the treatment required to achieve these outcomes, approximately $250 on average across the three sites per patient retained in care, was lower than reported in other studies from other low-income African countries [9,12]. Because published studies of treatment costs were conducted in a range of countries, covered different time periods, and used varying methods (detailed patient level data, facility-based averages, etc.) (see, e.g. [5,8,9,11,18]), comparing our results to those of studies from other locations is difficult. In Uganda, next door to Kenya, for example, the annualized cost of a newly initiated ART patient in 2009 USD was $967, more than three times our estimate. This difference was driven largely by the cost of antiretroviral drugs. Excluding ARVs from both cost estimates, however, the average cost of treatment in Uganda was still 54% more than in Kenya ($202 vs. $131). Although that study did not report outcomes, other studies from Uganda included in the review mentioned above [16] estimated 12-month retention rates of approximately 77%, slightly worse than those in our sample.

Although a 3% discount rate is commonly used in the public health literature (see, e.g. [9]), a 7% real discount rate was used to reflect the fact that investment capital in Kenya, and most other resource-constrained countries, is limited. Governments are unable to generate their own funds for development, which is why development assistance from other countries, including the US President's Emergency Plan for AIDS Relief (PEPFAR), supports investment projects. Because the majority of fixed costs at the sites were for annual site-level costs, such as salaries for staff not providing patient care, utilities, insurance on vehicles, and so on, rather than for purchases of equipment or buildings with long lives, the choice of the discount rate was not especially important for this analysis. Total fixed costs would fall by 2.5–5% across each site if a 3% discount rate was used instead of 7% (and 5–10% with a 0% discount rate).

Our study had several limitations. First, the number of study sites and the sample size at each site were small, suggesting caution in generalizing the findings to Kenya as a whole. Second, a number of potentially important costs arising from the provision of HIV treatment were excluded from the analysis. These include costs for care obtained outside of the study clinics, such as inpatient care. Programmatic costs incurred by the Ministry of Health, PEPFAR, and others for ART programme management were excluded. Although their omission makes our results an underestimate of the total cost of Kenya's national ART programme, our estimates are designed to be of direct budgetary and programmatic relevance to sites providing treatment and are methodologically comparable to other published estimates of the outpatient costs of ART [5,8, 18]. Third, information to evaluate quality of care, rather than just the quantity of resources used in patient care, do not exist. And fourth, for patients retained in care at 12 months, better information on their health status, such as viral load or CD4 counts at or near month 12 on treatment, do not exist largely because few tests are completed for patients at the sites.

Despite these limitations, we conclude from our findings that these sites in rural Kenya achieved good retention in care after 12 months on ART at a very low cost. The difference in total non-ARV costs between Kenya and Uganda mentioned above – for example, roughly $70 per patient – would translate into an annual savings to Kenya of some $30 million, enough to treat an additional 120,000 patients per year at the average cost estimated here. The size of ART programmes in Africa thus makes even modest cost savings per patient important at a national level.

On the other hand, our study also makes clear that there is little room left to reduce costs at sites such as these, barring further declines in ARV prices. The costs reported in Table 2 are for 2009 and are based on patient data accumulated between 2007 and 2009. Inflation and exchange rate differences would make these costs approximately 18% higher in 2012. Going forward, more changes can be expected. Most important, the annual cost in 2011 for d4T/3TC/NVP, the regimen most often used by our sample, was only $28/year. Although some 90% of patients in Kenya remained on d4T as of 2011 [15], the regimen now recommended by Kenyan guidelines, tenofovir/3TC/EFV, costs $172/year – six times more than the previous regimen. In an era of tightening budgets for ART programmes in Kenya and elsewhere, estimates such as those presented here make clear the trade-offs that policy makers will increasingly face between the quality of the treatment provided and the absolute number of patients who can be served [19].
